# Piloting a mHealth intervention to improve newborn care awareness among rural Cambodian mothers: a feasibility study

**DOI:** 10.1186/s12884-017-1541-z

**Published:** 2017-10-16

**Authors:** Shan Huang, Mu Li

**Affiliations:** 1 People in Need (Czech Republic), Prague, Czech Republic; 2 School of Public Health, The University of Sydney, Sydney, Australia

**Keywords:** mHealth, Feasibility, Pilot study, Maternal, Child, Newborn, Health, Nutrition, Voice messaging

## Abstract

**Background:**

Globally, the World Health Organization reports that the chances of a child dying is highest in the first month of life, the neonatal period. The neonatal mortality rate in Cambodia is 18 per 1000 live births. In the province of Kampong Chhnang, that rate is the fifth highest among the 24 provinces of Cambodia at 27 per 1000 live births. We piloted a project to determine the feasibility of using a mHealth intervention (the use of mobile devices to improve health outcomes) to increase mothers’ awareness about neonatal care and promote the government policy ‘Safe Motherhood Protocols for Health Centres’ which are in line with WHO recommendations for neonatal care.

**Methods:**

Between September and December 2013, we piloted an Interactive Voice Response system that sent pre-recorded messages to mothers of newborns using the theme ‘It takes a village to raise a baby’. Four hundred fifty-five mothers were registered onto this program and the intervention involved delivering seven periodic 60 to 90 s voice messages directly to the mobile phones of these mothers from day three of their neonate’s life to day 28. An evaluation of the pilot was conducted in December 2013. One hundred twenty-nine mothers were randomly selected from the 455 registered mothers and interviewed using a quantitative questionnaire. We also held two focus group discussions with three mothers and seven health workers.

**Results:**

Quantitative and qualitative results of 126 respondents were included for analysis. They indicate that the intervention was well accepted. Seventy-one percent of respondents reported that they would recommend the intervention to other mothers, and 83% reported that they would be willing to pay for the service.

**Conclusions:**

This type of mHealth intervention is an acceptable and feasible way of promoting the awareness of newborn care to rural Cambodian mothers.

## Background

Globally, the World Health Organization (WHO) reports that ‘*a child’s risk of dying is highest in the neonatal period of the first 28 days of life.* [1]’ It also reports that 45% of child mortality under five happens during the neonatal period [1]. The International Telecommunications Union (ITU) reports that by the end of 2015, there are more than 7 billion mobile phone subscriptions, reaching a 97% penetration rate [2]. Therefore, the use of mobile devices in maternal, newborn and child health (MCHN) programming has increased [3–5]. The ability of mobile phones to reach a wide audience on at relatively low cost makes it an attractive innovation for developing countries [3, 4, 6]. In the 2013 report by Philbrick W.C entitled ‘*MHealth and MNCH: State of the Evidence’*, the author reviewed mHealth interventions across the world and concluded that crosscutting approaches are needed to show the true impact of mHealth on MCHN outcomes [7]. It also reports that while what has been done is important, *how* programs are done are equally as important to understand the underlying social determinants of accessing quality services in mHealth [7]. In Cambodia, although 90% own a mobile phone [8], mHealth is still a novel concept with only a handful of projects being piloted with limited success, particularly in the area of using Short-Message Services (SMS) [9]. According to the latest Demographic Health Survey 2014, the neonatal mortality rate is 18 deaths per 1000 live births, even higher in the rural province of Kampong Chhnang where the rate is the fifth highest in the country at 27 deaths per 1000 live births [10]. We piloted a mHealth project in this rural province to ascertain the feasibility of a scalable program based on a contextual framework using an Interactive Voice Response (IVR) technology. The aim of this paper is to document how we designed the pilot mHealth project and results of whether or not it would be feasible to use a mHealth approach as a means for promoting timely information to improve mothers’ awareness regarding neonatal health in rural Cambodia.

## Methods

People in Need (PIN) – Czech Republic, a locally based International Non Government Organization (INGO), implemented a mHealth pilot in the local health administrative unit called the Kampong Tralach Operational District (OD) in Kampong Chhnang Province called ‘Baby Village Care’. It was designed to create more awareness regarding proper neonatal care in the first month of life based on what is recommended by the Ministry of Health Guideline for Safe Motherhood, issued in 2010 [11].

### Pre-pilot assessment

Between 1 and 19 July 2013, a small formative research study was done in the target area of Kampong Tralach OD in Kampong Chhnang Province. This consisted of three pre-pilot assessments. Firstly, two brief interviews with local health authorities at the OD and Provincial Levels regarding the status of recent neonatal mortality. Secondly, we undertook a pretested quantitative questionnaire on 78 randomly selected mothers with newborns to ask about their attitudes regarding of piloting an mHealth program. Thirdly, midwives in the target health administrative area were also interviewed using another pretested questionnaire at the health centre and referral hospital level regarding their perspectives of a mHealth intervention in this area. The results of the formative study fed into the design of the mHealth pilot.

### Design and description of the ‘baby village care’ program

**Fig. 1:**
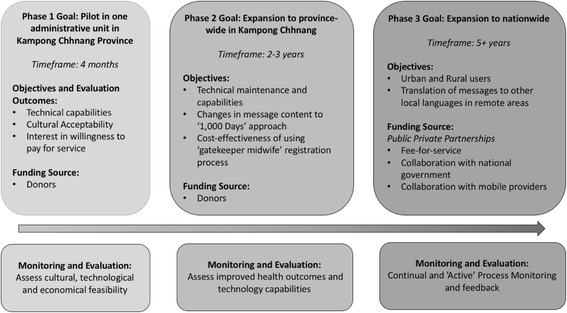
Conceptual Framework of the ‘Baby Village Care’ Program. Figure depicting the conceptual framework of the program discussed in the manuscript

The formative research results were incorporated into the design of the program. The ‘Baby Village Care’ program primarily targets mothers with newborns aged under 28 days who have access to a mobile phone. Our platform, an online cloud program entitled ‘Verboice’, used IVR technology to send seven voice-messages 60 to 90 s long directly to the mothers’ mobile phone starting from Day 3 of the newborn’s life. Verboice would call to the registered mobile phone number every 4 days until the newborn is 28 days old. Each message uses the different voices of several respected village ‘characters’ such as a doctor, a midwife, a village health worker, another mother, the village chief and an elderly grandmother. Given the novelty of mHealth programs, trained midwives at the health facilities were chosen to be the ‘gatekeepers’ of access to the service. Midwives from the target area received a half day interactive, hands-on training on how to register eligible mothers before the pilot was launched. Registration onto the service is done after the participating mother has given birth and before they are discharged from the health facility. We designed the pilot intervention with sustainability in mind and a conceptual framework was used to project future plans for the program that involved a model of self-funding from Public Private Partnerships. This is the unique feature of the pilot as often these are implement without a long-term goal in mind [12]. (Suggested placement of Fig. 1: Conceptual Framework).

### Pilot intervention and evaluation of ‘baby village care’

The pilot program occurred between 1 September to 31 December 2013 and 455 mothers registered. The National Ethics Committee of Cambodia approved the ‘Baby Village Care’ feasibility evaluation study of this pilot. All participating mothers who registered for the intervention gave their verbal consent to receive the automated voice messages. Recruitment and eligibility of the participating mothers were any woman who delivered their baby at a government Health Centre or the District Referral Hospital in Kampong Tralach OD who had the opportunity to have received all seven voice messages Table 1.

**Table 1:** List of voice messages and their topics

Message No.:	Message Topic:	Days after birth messages where automatically sent:	Example Message 4:
1	Cord Care: Taking care of the umbilical cord to prevent infection *Spoken by: Fictional Doctor (male voice)*	Day 3	Hey this is Midwife Sophea again calling from the Baby Care Village advice hotline! This is sad, but do you know that in Kampong Chhnang, baby death is about twice as high as the rest of the Cambodia? Together we need to change this. Here are some danger signs to watch out for: If your baby has difficulty breathing, convulses, has a fever, gets diarrhea, develops pus in its eye, or red blisters on the body, you should take your baby to the health center immediately. Don’t delay. Every second counts and waiting to go can be the difference between your baby’s life and death.
2	Exclusive Breastfeeding: Defined as breast milk alone is enough until baby is six months old, do not give even water or tea. *Spoken by: Fictional Village Health Volunteer who is also a mother (female voice)*	Day 7	
3	Effective Breastfeeding: Correct positioning and attachment *Spoken by: Fictional Midwife (female voice)*	Day 11	
4	Danger Signs: Go to seek care immediately if baby is bleeding, has continual diarrhoea, develops pus or red blisters *Spoken by: Fictional Midwife (female voice)*	Day 15	
5	Postnatal Care: ensuring mothers take post-partum iron and folate supplementation, reminder to get first vaccination for the baby *Spoken by: Fictional Doctor (male voice)*	Day 19	
6	Avoid Bad Behaviours: Do not smoke or drink alcohol when looking after baby *Spoken by: Fictional Village Chief (male voice)*	Day 23	
7	Keeping the baby warm: Baby should not be too hot or too cold. Mothers should not do ‘roasting’ – a traditional practice of sleeping in a sauna room *Spoken by: Fictional Elderly Grandmother (female voice)*	Day 28	
ALL	At the end of each message, there is a catch phrase: ‘*If you notice something wrong with your baby, take him/her to the local health facility right away*’.	ALL	

The evaluation of the pilot program took place from between 16 and 25 December 2013 with six trained enumerators, two of which also conducted and facilitated two Focus Group Discussions (FGDs). In the pilot evaluation, we interviewed 128 of the 455 participants using a pretested and back-translated quantitative questionnaire. These participants were randomly selected from the registered list of phone numbers recorded on the Verboice program and were interviewed over the phone. Two FGDs were conducted with one facilitator and one scribe. One FGD consisted of three mothers and three community health workers, the other consisted of all health workers (midwives and community health workers). The quantitative survey data was entered into SPSS version 20 and the results of the pilot evaluation were performed using SPSS version 22. Frequency analyses were mostly used to yield the results reported here.

## Results

For the pilot evaluation 128 mothers were interviewed using a quantitative questionnaire. Their general characteristics were an average age of 27 years, all but two were married, 47% were first time mothers, 70% had access to their own mobile phones (mobiles that belonged to the participant and not to their husband or other family members) Table 2.

**Table 2:** Demographics of Participants in the Pilot Evaluation

Area of Demographic	Results:
Ownership of mobile phone	70% (*n* = 90)
Age	Average = 27 years (Range: 17-41 years)
Marital Status	Married: *n* = 126, Widowed: n = 2
Parity	First time mothers = 61, Range 1-6
Number of people in the Household	Average = 5 (Range: 2-14)

### Acceptability

A key aim of the pilot intervention was to assess whether or not this type of intervention would be acceptable to the target audience and thus feasible for potential scale-up. The results indicate that acceptability was high with all mothers listening to at least four of the seven messages. Sixty-one percent mothers surveyed report that they told others about the service, 71% would recommend the service to other mothers and 63% reported sharing the information they heard with others. The results from our FGD with the health workers also responded well to the program. They report that the program was a good way of connecting with mothers, as one midwife said ‘*This (initiative) makes mothers more aware of how to take care of (their) babies’*. Our quantitative results indicate that 85% of the mothers surveyed listened to some messages not just once but several times, using the option in the IVR to repeat again, allowing the messages to be heard by other members of the household such as their husbands (*n* = 87), mothers (*n* = 67), siblings and older children (*n* = 23). The FGD results also showed that some first-time mothers said they had ‘*shared the messages for other members of the household to listen to’*.

Further, 96% of respondents reported the length of the messages (60 to 90 s) was appropriate and all respondents reported that the intervention was helpful for them when looking after their newborn. Additionally, 45% also reported learning something new from listening to the messages. The majority of respondents (96%) report that they would like to see the service extended, 44% of which suggested a service extension of at least 12 months. Finally, results also showed that 43% of mothers reported that they took their babies to the health centre as a result of listening to the messages.

### Technology

We also assessed a number of technological points to judge user satisfaction. The timing of the automated calls (between 1800 and 2000 h, as suggested during the pre-pilot formative research) was one aspect of importance because we wanted to target a time when the mother is at home to receive the message and increase the chances of the messages being heard. Our evaluation results indicate that 36% of respondents received a call during the preferred time slot and the majority received calls at other times of day. Despite this, only 6 of those surveyed reported that they thought the timing of calls was inconvenient. The main reasons for inconvenient call time were that they did not have their phone with them at the time of the call or they were too busy to answer the call. The technical aspects of line rental provided by the private telecommunications company was also assessed (i.e. call clarity, sudden cut-offs). All respondents surveyed reported the line was clear and only 12 out of those surveyed reported their call was cut off while listening to the messages. Discussion during the FGDs included one first-time mother mentioning that *‘At anytime (when I was listening to the message) the line was clear. The timing was convenient for me because normally mothers with new babies stay home after birth and do not go anywhere’*.

### Willingness to pay

To make this type of mHealth intervention financially sustainable willingness to pay was assessed. A majority (60%, *n* = 75) of respondents said that they would be willing to pay for the service. The fee ranged being between 100 and 3000 Cambodian Riels (4000 Riels = 1 USD) with an average willingness to pay of 577 Riels. One mother from the FDG remarked *‘I would like to pay USD 0.10 for the messages because they are very important to child health’.* Despite this willingness to pay reported from the mothers, it should be noted that during the FDG with the midwives and community health workers, they reported that the program should be made free to mothers as they suspected that ‘*most mothers do not have enough credit on their phones (to pay for the messages)*’.

## Discussion

### IVR versus SMS

In this pilot study, we established that a mHealth intervention using IVR technology is able to engage mothers and families in neonatal health care in Cambodia. Despite the popularity of using SMS as part of a mHealth program, particularly in Africa as reminder services [13, 14], we chose to use the IVR platform to send voice-messages to empower mothers with health knowledge. This is due to the fact that a recent local report showed that only 29% of the mobile phones in Cambodia support the local Khmer script [8]. Couple this with low levels of literacy among rural women, voice-messaging was the better option for this intervention. A similar program by Crawford et al. in Malawi, indicated that there was no difference in the effectiveness between either IVR or SMS modalities [15]. It reports that both methods can be highly effective and supported by the target users [15].

### Contextual feasibility

One of the criticisms of mHealth projects is that they lacked contextual framework [6, 12]. Contextualising the program into the intervention’s design ensures a greater chance of program success [16, 17]. We implemented our pilot within our conceptual framework (see Fig. 1). The pilot was the first piece of a bigger puzzle that part of a bigger plan to build a sustainable program based on cultural, financial, and technological solutions. Therefore, the results of the pilot evaluation will be used as a base for the next phase (currently underway) which will include buy-in from high level government stakeholders as well as private partners (for example mobile telecommunication providers). This kind of enabling environment from the get-go can be the foundation on which the interventions like this can be scaled up in a step-by-step approach.

### Cultural feasibility

The cultural aspect of our intervention was the content of our voice-messages. The dramatic ‘role play’ style meant that listeners trusted the messages which were inspired by the successful Aponjon Program in Bangladesh [18]. There, a similar type of dramatic style was used to build rapport with listeners, facilitating a supportive environment for the intervention’s acceptance [18, 19]. It has been criticized that mHealth initiatives in developing country contexts are more often than not, developed in English rather than the local language [12]. However, in our case, we developed the text in both English and Khmer languages simultaneously. The Khmer version then field-tested to ensure the correct cultural meaning was relayed and any nuances were well understood. The outcome that mothers were happy to recommend the service to other mothers is a key demonstration of trust in the program and the information it provided. Additionally, other household members also listened to the messages due to a repeat function in the IVR pathway. Results show that these ‘other listeners’ were often the husband, mother and sibling of the primary listener. In Cambodian culture, the primary decision holder is the head of the household, usually the husband. However, history has suggested that the Khmer culture follows a matriarchal system [20], hence the maternal grandmother (who often lives with the family) also has great decision-making power, particularly with regards to child rearing. This is likely to have a significant influence on the empowerment of the mother, especially on issues of baby care if all three (the targeted mother, her husband and her mother) are in agreement to the same information and action. Matsuoka et al., showed that in their qualitative research, the beliefs and opinions of the Cambodian family elders played a significant psycho-cultural role in the decision making power of the mother to seek professional medical care [21]. While women and mothers are often the target of mHealth interventions, particularly around maternal and child health, it is also crucial for programs to offer the chance to communicate with men and the youth to support a family style approach to see real behaviour change. Our method to include both male and female respectable characters into the messages is likely to have had an impact on the believability of the messages. This correlates with our results that indicate the intervention was able to prompt mothers to take the baby to the health facility as a result of listening to the messages.

### Technological feasibility

While there are high phone ownership rates in Cambodia, there is also the issue of owning more than one SIM card. In fact, more often than not, ownership of up to four SIM cards, all on different networks has been reported [8]. This is great challenge when the mHealth intervention is only registered on one SIM. There are four major telecommunication providers in Cambodia and SIM card switching is a frequent occurrence and a hindrance to interventions such as ours where only one number from one network is registered [9]. While our feasibility study reports that telecommunication lines were clear with only some calls being missed, having more than one SIM is one of the biggest barriers to the end-user’s ability to access all the messages in a timely manner. One of the limitations of our evaluation was that we did not ask whether or not the number used for registration was the ‘primary’ number being used and if indeed our target audience switched SIM cards.

### Economic feasibility

One major limitation to a mHealth program’s sustainability is its funding [3, 6]. Some types funding arrangements (namely donor funding) can predispose the systems to sustainability challenges [6]. While a large majority of the mHealth pilots and scale-up interventions are being implemented using donor funding [14], we wanted to create a program that would eventually fund itself. Though our initial pilot was free, the fact that our participants want the program extended and were willing to pay a small fee for it, suggest that they see the value in the content of the messages. This echoes the findings of the Aponjon program’s formative research report where 76% and 66% of pregnant women and new mothers respectively said they were willing to pay a ‘small fee’ for the service [22]. However, the amount users were willing to pay was much higher in our context (0.01 USD versus 0.13 USD). Unlike traditional preventative and curative health services, the addition of fees can abruptly decrease the utilization of the service or quality of service [23, 24]. Our results indicate that the willingness to pay has the potential to engage further utilization without compromising on the quality. This is because the quality of the content of voice-message is controlled by the implementing NGO, not a third party. mHealth is a very new concept to rural Cambodian mothers and to our knowledge, this was the first intervention of its kind in Cambodia. For there to be a willingness to pay for a service like this could mean a great potential to scale-up and may see real health impacts if the enabling environment is supported (socially, economically, technologically). However, the downside to a fee-based approach would limit the program to those who are willing and able to pay, thus neglecting the poorest of the poor who may be unable to do so. We envisage that the program would be offered for free to those who have an ID Poor card (a government concession card given to those who meet a set of poverty criteria). To pay for these users, based on our conceptual framework, we expect a public private partnership could be an ideal way for any fee off-sets and scale up in the future. The intention of a user-paid service was to not devalue the messages. The idea of offering the program free for everyone may be more equitable, but if people were willing to pay for something, it honours their higher commitment and value to what is being purchased.

### Strengths

A strength of this feasibility study is its bottom-up approach guided by a conceptual framework. It uses a family theme to be inclusive of not just mothers but their husbands, family elders and siblings. The pilot also considered social-political norms through early engagement with local authorities and using the existing health infrastructure as well as considering cultural and environmental influences.

### Limitations

The intervention period of four months is short and the exposure time to the intervention was four weeks. The results are likely to indicate some recall bias depending on how well mothers remember the intervention compared to when they were surveyed for the pilot evaluation. For example, some respondents recall receiving ten messages. Given there are only seven messages in total, this could be deduced to recall error or a technical issue where some messages were sent more than once (neither was investigated for in this instance). Finally, we did not collect any information on the literacy, education level or social economic factors of the targeted mothers. Such factors may have influenced the outcome of the pilot intervention. Our study focused on the feasibility of a mHealth intervention of this kind, however, we did not have the resources to assess the effects of the intervention on health outcomes. Our messages were also only available to mothers who delivered their children in a health facility, which meant that those who still delivered at home who maybe more likely to be a poorer, more vulnerable group are still at risk.

### Recommendations for similar mHealth or mobile technology interventions in other settings

For others considering implementing a similar project in other settings, we recommend that detailed pre-pilot work be considered to assess the community and local authority buy-in to the mHealth idea if it is novel. The results of this can feed into the design of the program and can be of valuable importance. During the development of the program content, based on our experience and results, the more realistic the messages can be, the more likely the target audience will be to responding to them. In our case, we tried as much as possible to use ordinary local wording, spoke clearly and slowly, using ‘trusted’ voices such as a village chief, a midwife, a peer mother etc. Though our pilot targeted mothers with infants, a largely female audience, we did not limit our messages to women only, but also reached out to men as they are a key part of the decision making process. As such, for other programs wanting to use IVR technology, we would suggest considering messages (their content and ‘voice’) to be as broad-reaching as it is possible, as it was in our case, so that those other than the targeted mobile user may also be listening in. However, on the flip side, if the program being implemented is to discuss highly private content (such as issues of domestic violence), consideration will need to be given to the potential that there may be other secondary users of the mobile. Finally, depending on the technology platform being used, try to make the system as user-friendly as possible. Consider putting fail-safes in place wherever possible so that if a mistake were to be made, there would be an option redo for the user.

## Conclusion

This pilot study was effective in justifying that this type of mHealth intervention, using IVR technology, is an acceptable way of improving the mothers’ awareness of newborn care. The results indicate that while this type of intervention is feasible on technological, cultural and economic grounds, more studies need to be done to show the impact of messages on behaviour change that can lead to improved MCHN outcomes through expansion to over the 1000 days period.

## Abbreviations

FDG: Focus group discussions
INGO: International Non-Government Organization
ITU: International telecommunication union
IVR: Interactive voice-response
MCHN: Maternal Child Health and Nutrition
NGO: Non-Government Organization
OD: Operational district
PIN: People in need
SMS: Short messaging service
USD: United States dollar
WHO: World Health Organization
